# Protocol for quantitative analysis of adult zebrafish swimming behavior using DeepLabCut

**DOI:** 10.1016/j.xpro.2026.104374

**Published:** 2026-02-17

**Authors:** Pramuk Keerthisinghe, Anwarul Karim, Aditya Basak, James P. Orengo

**Affiliations:** 1 Department of Neurology, Baylor College of Medicine, Houston, TX 77030, USA; 2 Rice University, Houston, TX 77005, USA; 3 Department of Neuroscience, Baylor College of Medicine, Houston, TX 77030, USA

**Keywords:** Model Organisms, Neuroscience, Behavior, Computer sciences

## Abstract

Here, we present a protocol for quantifying adult zebrafish swimming behavior using open-source software. We describe steps for recording and preprocessing swimming behavior videos, labeling and training a DeepLabCut network, and applying the trained model to analyze behavior. We then detail procedures for processing and visualizing behavioral data. This protocol is optimized for single-fish tracking under standardized recording conditions and can be completed using beginner-level computational skills with basic laboratory hardware.

## Highlights

• Reproducible workflow for recording and analyzing adult zebrafish swimming behavior
• Pretrained DeepLabCut model enables immediate analysis by beginners
• Step-by-step guidance for training and validating new DeepLabCut models
• Smartphone-based recording setup using standard zebrafish tanks

## Abstract (editor highlights)

Publisher’s note: Undertaking any experimental protocol requires adherence to local institutional guidelines for laboratory safety and ethics.

## Graphical abstract

**Figure:**
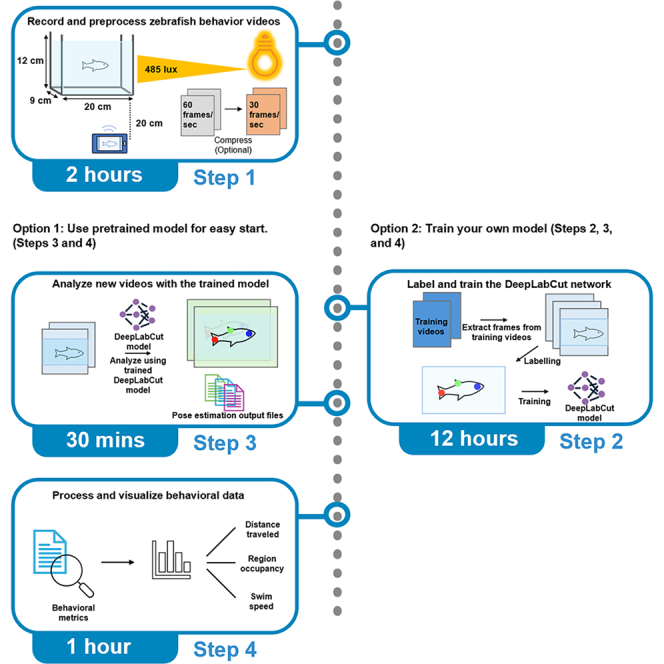

## Before you begin

### Experimental setup

**Figure 1:**
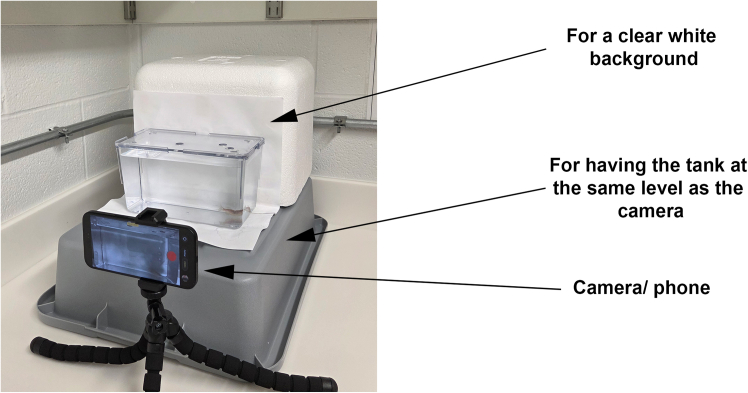
Recording setup showing tank dimensions, camera position, and lighting configuration
4. Place a white paper background behind the tank to improve contrast.
5. Maintain ambient room illumination (∼485 lux) and record at 30 fps (preferred) or 60 fps 4K (optional for higher precision).
6. Record a single adult zebrafish per session.

***Note:*** Adult zebrafish aged 5–16 months were used; the pretrained model is validated across this range.

***Note:*** Recordings were performed on single, wild-type fish of mixed sex (standard length ∼2.5–4 cm).

1. Recommended to use of clear Aquatic Habitat tanks (20 cm L × 12 cm H × 9 cm W; ≈1.8 L).
2. Fill tanks to the top with clean system water (recirculating zebrafish facility water maintained under standard husbandry conditions; pH ∼7.0–7.5, conductivity ∼300–500 μS).
3. Mount the camera (minimum 1080p resolution at 30 fps; higher resolutions such as 4K recommended) on a tabletop tripod 20 cm directly in front of the tank, as shown in Figure 1.

7. Allow each fish to acclimate for at least 5 min before recording.

**CRITICAL:** Maintain consistent lighting and camera positioning across all recordings to ensure tracking accuracy.

***Note:*** Although the general workflow may be adaptable, this protocol has been specifically optimized and validated for adult zebrafish, and it replicates established zebrafish locomotor paradigms.^1^

### Computational setup

DeepLabCut v3.0.0 rc6 (Mathis Laboratory) was installed in a conda environment configured for GPU acceleration using PyTorch v2.5.1 and CUDA 12.1.

All analyses were performed using a conda-managed environment (Anaconda v24.1) on a Lenovo laptop equipped with an NVIDIA GeForce RTX 3060 Laptop GPU (6 GB VRAM) running Windows 10. Note: MacOS and Linux systems are compatible with minor modifications.

The trained network achieved a mean training error of 9.9 pixels and a mean test error of 10.4 pixels (pcutoff = 0.6).

Mean average precision and recall were 76.0%/79.9% for training and 74.9%/78.1% for testing.

The analysis scripts provided with this protocol are optimized for CUDA-enabled NVIDIA GPUs. While DeepLabCut can be run on CPU-only systems, non-CUDA execution may require additional configuration and substantially longer runtimes, which may be challenging for users with limited Python experience.

### Innovation

This protocol integrates the DeepLabCut open-source framework with a standardized zebrafish behavioral recording pipeline to enable accurate single-fish tracking. The workflow minimizes ID-swapping by focusing on individual fish within a controlled tank environment and includes a pretrained model validated across multiple age groups (5–16 months). This protocol allows even novice users with a standard computer and freely available software to begin quantitative analysis of adult zebrafish swimming behavior immediately.

### Rationale

Quantitative assessment of adult zebrafish swimming behavior is critical for studying motor coordination, ataxia, and neurodegenerative disorders.

Although several behavioral tracking tools are available, such as Ethovision and idTracker, these systems often require commercial license, strict lighting conditions, or high-contrast backgrounds to achieve reliable tracking.^2^ In contrast, open-source deep learning frameworks like DeepLabCut offer markless tracking but typically demand advanced computational setup and training knowledge.^3^^,^^4^

This protocol provides a simplified, reproducible, and freely accessible workflow for researchers aiming to quantify zebrafish behavior without specialized coding expertise. It combines a standardized, low-cost recording configuration with a pretrained DeepLabCut model optimized for adult zebrafish, while still supporting optional model retraining for users who wish to adapt it to their own experimental conditions.

### Institutional permissions (if applicable)

All zebrafish behavioral procedures were performed in accordance with institutional animal care and use guidelines at Baylor College of Medicine. Animal handling and recording protocols were reviewed and approved by the Institutional Animal Care and Use Committee (IACUC) at Baylor College of Medicine. Investigators should obtain equivalent approval from their institutional regulatory body prior to performing animal experiments described in this protocol.

## Key resources table

**Table:** 

REAGENT or RESOURCE	SOURCE	IDENTIFIER
**Deposited data**		

Pretrained DeepLabCut model and analysis scripts	This study	https://doi.org/10.5281/zenodo.17624893

**Experimental models: Organisms/strains**		

*Zebrafish* (*Danio rerio*), wild-type adult	Baylor College of Medicine Zebrafish Core	Wild-type; age 5-16 months; sex mixed

**Software and algorithms**		

DeepLabCut software	Mathis Laboratory	v3.0.0 rc6; https://www.deeplabcut.org
PyTorch	PyTorch Foundation	v2.5.1 + CUDA 12.1
Python	Python Software Foundation	v3.10.16
FFmpeg	FFmpeg Project	https://ffmpeg.org
Anaconda (Conda environment manager)	Anaconda, Inc.	v24.1
Microsoft Excel	Microsoft	Microsoft 365
Python analysis scripts	This study (Zenodo)	https://doi.org/10.5281/zenodo.17624893

**Other**		

Aquatic Habitat tanks (1.8L)	Pentair Aquatic Eco-Systems	Model #AH-TNK1.8
iPhone 15 camera	Apple Inc.	Native camera app, 30-60 fps
Tabletop tripod	Amazon basics	Adjustable tabletop tripod for camera mounting
White paper background	Local stationary supplier	White paper used as imaging background
Light meter (LT300)	Extech Instruments	Digital lux meter used for ambient light calibration
Laptop computer	Lenovo	NVIDIA GeForce RTX 3060 Laptop GPU, 6 GB VRAM, Windows 10, minimum of 16 GB system RAM

## Step-by-step method details

### Overview of options for using this protocol

This protocol can be followed in two ways depending on the user’s needs and computational experience.

1. Option A: Use the pretrained model (recommended for beginners).

This option allows users to directly analyze adult zebrafish swimming videos using the pretrained DeepLabCut model provided by the authors. Users should follow the sections record and preprocess videos, analyze videos using the trained model, and process and visualize behavioral data. No network training is required.

2. Option B: Train a new model.

Users with different lighting conditions, tank configurations, or experimental design may choose to train a new DeepLabCut model. In this case, users should follow all sections of the protocol, including label and train the DeepLabCut model, before proceeding to video analysis and data processing.

***Note:*** Both options share the same recording setup, video preprocessing steps, and downstream analysis scripts. Users can select the most appropriate path based on their computational resources and experimental goals.

### Record and preprocess videos

**Timing: 1–2 h**

**Figure 2:**
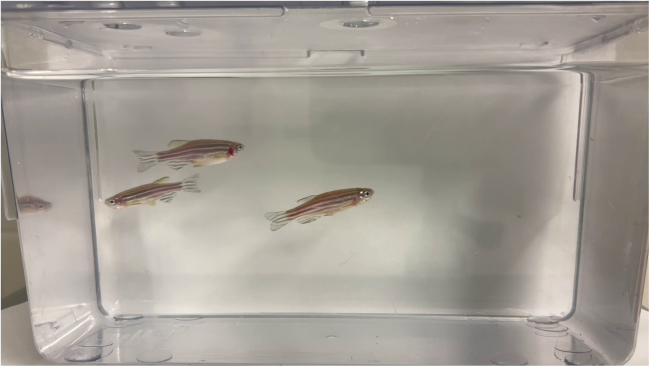
Example frame showing multiple fish used during model training and illustration only; protocol performance is optimized for single-fish recordings

Here, we describe how to record adult zebrafish swimming behavior under standardized conditions (Figure 2) and prepare videos for DeepLabCut analysis.

3. Place a single fish in the tank and start recording behavior.
4. Save videos in MP4 or MOV format at the specified frame rate.
5. Trim videos to the desired duration and rename using consistent identifiers.
6. Convert files to 30 fps if high-speed recordings slow analysis.

**CRITICAL:** Maintain consistent lighting and camera position for all sessions.

***Note:*** Poor lighting (<300 lux) can reduce tracking accuracy.

### Label and train the DeepLabCut model

**Timing: 4–12 h (depending on training iterations and hardware)**

Here, we describe how to extract frames, label keypoints, and train a DeepLabCut network for adult zebrafish swimming behavior.

***Note:*** If you are using the pretrained model provided by the authors, skip this step and proceed directly to Step 13.

**Figure 3:**
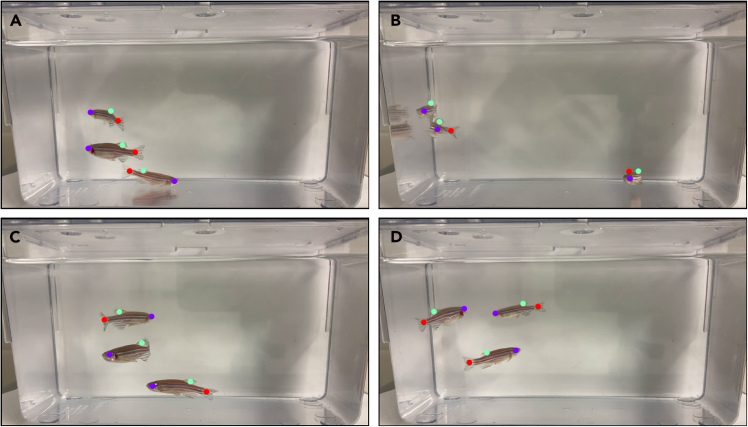
Example of manually labeled frames in DeepLabCut GUI showing keypoints (eye, dorsal fin, caudal fin) Example multi-fish recordings used during the training phase of the DeepLabCut model. Although multiple fish are shown here to illustrate training diversity, the protocol is optimized for single-fish behavioral analysis during experimental recordings.
10. Configure config.yaml to match project settings (e.g., network type = ResNet-50).
11. Train the network on GPU until the error plateaus (∼1.5 h).

***Note:*** Training was performed in DeepLabCut v3.0.0rc6 PyTorch and Anaconda environments. The workflow follows the supervised learning approach described by Mathis *et al.*^3^ and further refined for 3D and multi-animal tracking in later versions.^4^^,^^5^

7. Import 16 representative videos into DeepLabCut.
8. Extract 20 frames per video for manual labeling.
9. Label three key points: eye, dorsal fin, and caudal fin - as shown in Figure 3.

12. Evaluate the trained model to confirm test RMSE ≈ 10 px.

**CRITICAL:** For training, visually distinct fish were selected to improve model robustness. These included differences in body size, fin length, and pigmentation patterns (e.g., variation in dorsal or caudal fin shape and overall body contrast), ensuring the network was exposed to diverse body morphologies.

***Note:*** If GPU memory is insufficient, reduce batch size to 1 in the config file.

During model training, videos containing three fish were used to increase pose diversity and robustness. However, all downstream behavioral quantification described in this protocol is performed on single-fish recordings to avoid ID swapping.

**Pause point:** The trained model can be stored and reused for future datasets.

### Analyze videos using the trained model

**Timing: 10–30 min per video (GPU dependent)**

Here, we describe how to use the pretrained or newly trained DeepLabCut model to perform pose estimation on adult zebrafish swimming videos.

**Figure 4:**
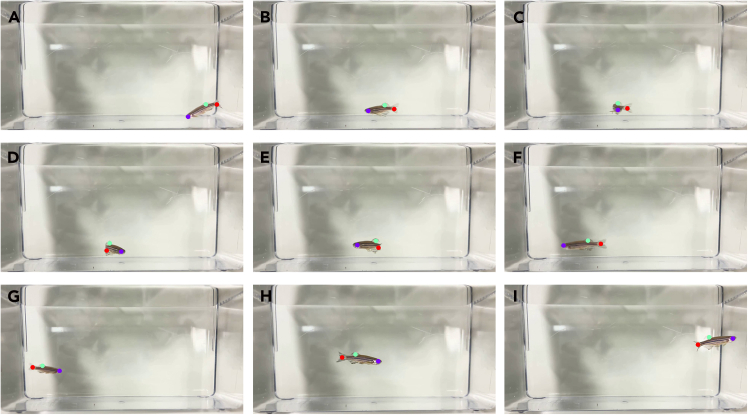
Tracking overlay showing predicted keypoints during analysis step

13. Load the trained network and select target videos for analysis.
14. Run pose estimation to generate CSV/HDF5 output containing x-y coordinated and likelihood for each keypoint per frame.
15. Inspect tracking overlays to confirm correct identification, as shown in Figure 4.

**CRITICAL:** Ensure video frame rate matches training data to avoid temporal drift.

***Note:*** An example annotated output video showing pose estimation and tracking overlay is provided as Methods Video S1.

Methods Video S1. Example output video showing single adult zebrafish tracked using the pretrained DeepLabCut model, related to Step 13

### Process and visualize behavioral data

**Timing: 10–30 min per dataset**

**Figure 5:**
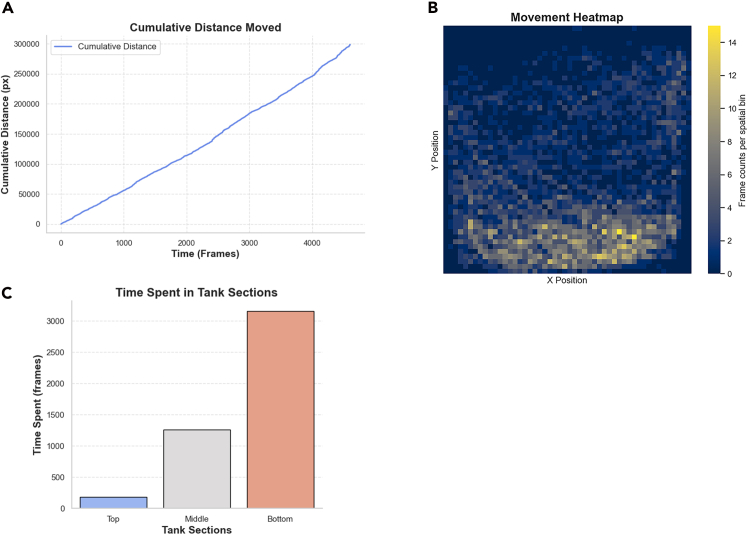
Example behavioral metrics derived from DeepLabCut tracking Example output plots: (A) distance traveled over time, (B) heat map of spatial occupancy, (C) proportion of time spent in top/middle/bottom regions. In the heat maps, color intensity corresponds to the number of video frames spent in each spatial bin.

Here, we describe how to compute behavioral metrics and generate plots such as trajectories, distance traveled, zone occupancy, and heatmaps (Figure 5).

16. Import DeepLabCut output files into the provided Python analysis script.
17. Compute behavioral metrics:
  a. Cumulative distance traveled (pixels).
  b. Zone occupancy (top/middle/bottom regions).
  c. Average swim speed (pixels per frame).
  d. Heat map of spatial distribution.
  e. Likelihood score plots (generated using the provided analysis script; users may enable or disable likelihood visualization depending on their analysis needs.) Likelihood values are included in the output CSV files and can be plotted using the provided Python script.
18. Export summaries as CSV or Excel files.

***Note:*** Minor missing frames can occur if the fish briefly leaves focus.

## Expected outcomes

In well-lit 30 fps recordings, the trained model tracks fish accurately in nearly all frames. Tracking occasionally fails only when the fish leaves focus or becomes blurred. The resulting output files include positional data, likelihood values, and annotated videos suitable for quantitative behavioral analysis. This protocol yields accurate pose tracking of individual zebrafish across almost all frames. Outputs include tracked videos, CSV/Excel files of keypoint coordinates, and derived behavioral metrics. The achieved test error (≈10 pixels) is consistent with previously reported DeepLabCut performance for animal pose estimation.^3^^,^^4^ On GPU-enabled systems, analysis of standard 30 fps videos typically completes within minutes, whereas CPU-based execution (if manually enabled) may require substantially longer runtimes.

## Quantification and statistical analysis

DeepLabCut outputs were processed in Python and Microsoft Excel to extract quantitative behavioral parameters. Each keypoint (eye, dorsal fin, caudal fin) produced x-y coordinate time series per frame from which the following metrics were computed.

• Distance traveled (pixels): cumulative displacement per frame.
• Zone occupancy (%): proportion of frames spent in top, middle, or bottom tank regions.
• Average speed (pixels/frame): first derivative of position with respect to time.
• Heat map: representation of the absolute number of frames in which the fish occupied each spatial bin of the tank across the recording duration.

Data were summarized as mean ± SEM per fish or per experiment group. Tracking quality was evaluated by mean RMSE (train = 9.9 px; test = 10.4 px) and keypoint detection likelihood (cutoff 0.6). Statistical comparisons between groups (if applicable) can be performed using one-way ANOVA or t-tests on derived behavioral metrics such as total distance or zone occupancy. When using parametric tests, users should first confirm that the data meet the assumptions of normality and homoscedasticity; otherwise, appropriate non-parametric alternatives should be considered.

## Limitations

Tracking accuracy decreases with motion blur or dim lighting (<300 lux). Multi-animal tracking can result in identity swapping if more than one fish is present, as previously noted in multi-animal DeepLabCut implementations.^5^ High-framerate 4 K videos require more GPU resources for analysis. The analysis scripts provided with this protocol are configured for CUDA-enabled NVIDIA GPUs. Users without access to CUDA-capable hardware may encounter errors when running the code and would need to manually modify the scripts to enable CPU-based execution, which may be challenging for users with limited Python experience.

## Troubleshooting

### Problem 1

GPU not detected during analysis (Step 14).

### Potential solution

It is possibly caused by CUDA/PyTorch mismatch. Verify CUDA toolkit version and update drivers. This protocol and provided scripts are configured for CUDA-enabled NVIDIA GPUs. On CPU-only systems, DeepLabCut may require additional manual configuration (e.g., modifying configuration files to disable CUDA support), which is beyond the scope of this protocol.

### Problem 2

Poor tracking during analysis (Step 15).

### Potential solution

It may be as a result of low lighting or blurred videos. Increase illumination or frame rate.

### Problem 3

Training crashes (Step 11).

### Potential solution

Batch size may be too large. Reduce batch_size to 1 in config file.

### Problem 4

Missing output files (Step 15).

### Potential solution

It could be due to incorrect paths. Check project directory and file permissions.

### Problem 5

Model fails to load during analysis (Step 13).

### Potential solution

It is primarily due to wrong snapshot file. Use latest ∗_snapshot_200 model checkpoint.

### Problem 6

Tracking output misaligned with video playback (Step 15).

### Potential solution

Ensure that the analyzed video frame rate matches the original recording frame rate.

## Resource availability

### Lead contact

Further information and requests for resources and reagents should be directed to and will be fulfilled by the lead contact, James Peter Orengo (james.orengo@bcm.edu).

### Technical contact

Technical questions on executing this protocol should be directed to and will be answered by the technical contact, Pramuk Keerthisinghe (pramuk.keerthisinghe@bcm.edu).

### Materials availability

No new biological materials were generated.

### Data and code availability

Pretrained DeepLabCut model, analysis scripts, and example datasets will be made available on Zenodo with https://doi.org/10.5281/zenodo.17624893.

## Acknowledgments

This work was supported by a Baylor College of Medicine Seed Grant and the Mrs. Clifford Elder White Graham Endowed Research Award. The authors thank the Baylor Zebrafish Core Facility for animal husbandry and technical assistance. We also thank members of the Orengo laboratory for helpful discussions during model development and testing. P.K. acknowledges guidance from J.P.O. in project supervision and support.

## Author contributions

Conceptualization, P.K. and J.P.O.; methodology, P.K. and J.P.O.; software, P.K.; investigation, P.K.; data curation, P.K.; visualization, P.K.; writing – original draft, P.K.; writing – review and editing, P.K., A.K., A.B., and J.P.O.; supervision, J.P.O.; funding acquisition, J.P.O.

## Declaration of interests

The authors declare no competing interests.

## Declaration of generative AI and AI-assisted technologies in the writing process

During the preparation of this work, the authors used *ChatGPT* (*OpenAI*, *San Francisco*, *USA*) to assist in language refinement and organization of the manuscript. After using this tool, the authors reviewed, edited, and verified all content and take full responsibility for the final version of the manuscript.
